# P-190. Enhancing *Clostridium difficile* Diagnosis and Management: A Structured Approach to Early Recognition with Two-Step Testing

**DOI:** 10.1093/ofid/ofae631.394

**Published:** 2025-01-29

**Authors:** Muhammad Yasser Alsafadi, Shivani Patel, Firas Zabaneh, Ashley Drews

**Affiliations:** Houston Methodist, Houston, Texas; Houston Methodist, Houston, Texas; Houston Methodist, Houston, Texas; Houston Methodist, Houston, Texas

## Abstract

**Background:**

*Clostridium difficile* is a significant challenge in healthcare settings, requiring accurate and timely diagnosis while avoiding unnecessary treatment. To reduce false positives, IDSA/SHEA recommends two-step testing: a sensitive assay to detect *C. difficile* followed by a specific assay to confirm toxigenic CDI. Our longstanding CDI prevention program, with a comprehensive testing algorithm, consistently outperformed the national NHSN benchmark. This CDI infection prevention program is multifaceted, including early recognition, appropriate testing strategies, accurate attribution, and stewardship interventions. Here, we evaluated the performance of two-step testing on identifying patients with discordant results and related antimicrobial consumption.

**Figure:**
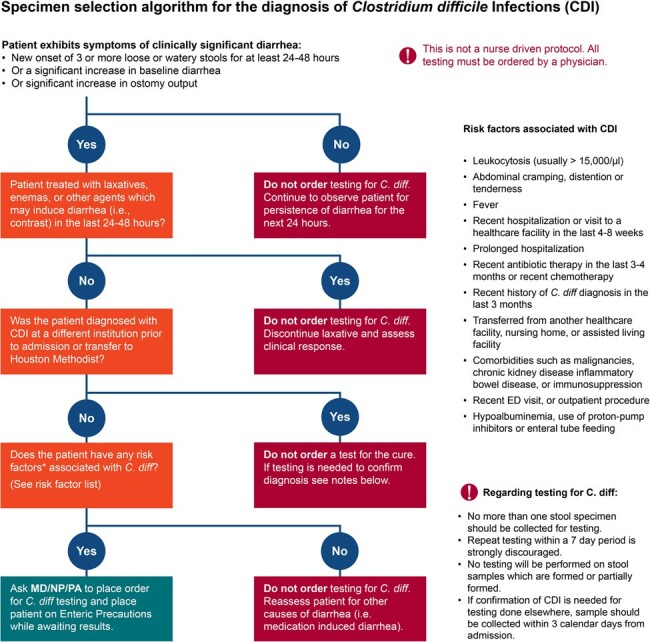

**Methods:**

In December 2023, we updated our CDI prevention program with enhanced focus on early detection using a testing selection algorithm (Fig 1) as well as two-step testing (Fig 2) including a *tcdB* PCR assay and a toxin enzyme immunoassay to identify discordant results (PCR+/toxin-) that usually do not require treatment. We assessed *C. difficile* testing and treatment at all 8 hospitals in our system and hospital-onset (HO)-CDI at the flagship hospital from January to March 2024.

**Figure:**
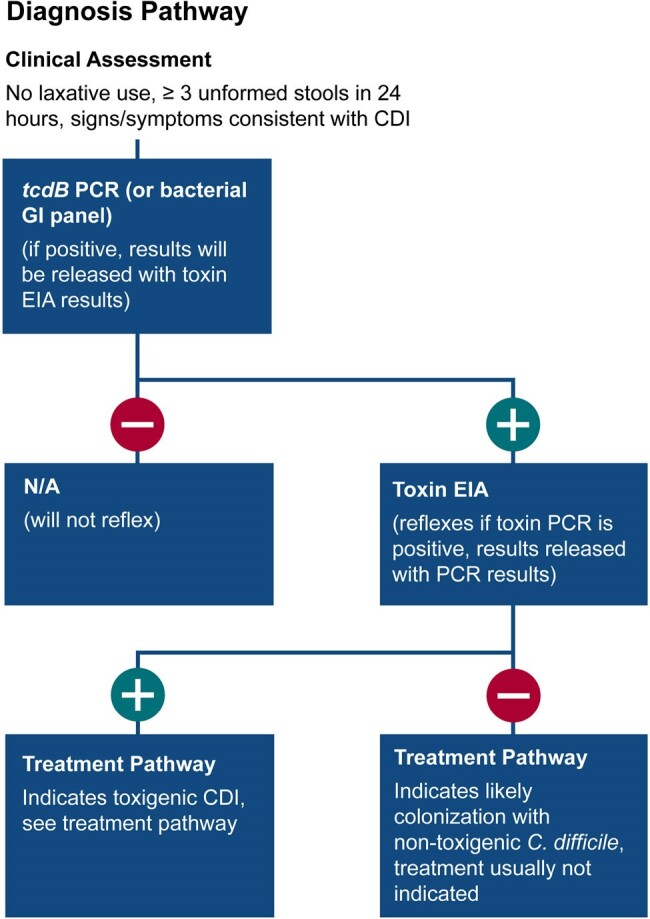

**Results:**

From January 1 to March 31, 2024, 1,864 patients were tested for CDI by PCR assay; of the 328 that were positive, 111 (33.8%) were also positive for toxin (+/+), with the other 217 (66.2%) negative for toxin (+/-) representing non-toxigenic colonization for which treatment is not recommended. Out of 1,428 days of treatment given for CDI, 274 and 574 were in patients with negative (-/-) and discordant (+/-) results, respectively, indicating that treatment is still prevalent in these groups despite recommendations. HO-CDI rates decreased, with the NHSN standardized infection ratio significantly lower in March 2024 than in 2023 (Fig 3).

**Figure:**
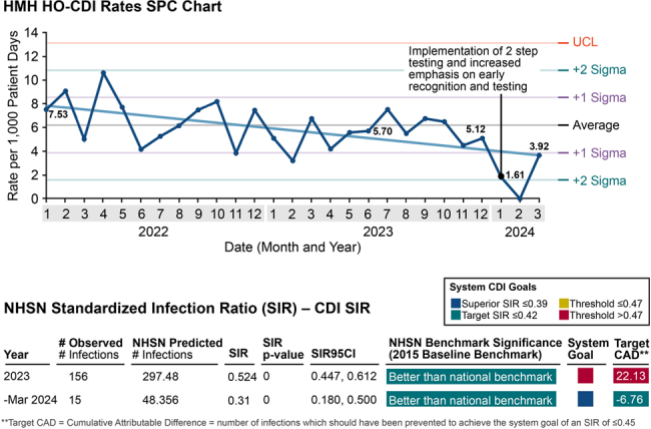

**Conclusion:**

In 2024, we observed a reduction in reportable HO-CDI across the health system. Additional stewardship interventions will be required to decrease treatment in patients with discordant results. This will be of increased importance as NHSN evaluates and updates changes to definitions around reportable CDI.

**Disclosures:**

**All Authors**: No reported disclosures

